# Analysis of Targeted Post-operative Nursing Outcome in 1246 Patients with Percutaneous Transhepatic Biliary Drainage

**DOI:** 10.3389/fsurg.2022.908909

**Published:** 2022-04-27

**Authors:** Xiuchun Yang, Yuelan Qin, Wei Mo, Hua Xiang, Zhichao Liu, Jianhua Long, Bin Xiang

**Affiliations:** ^1^ Department of Interventional Vascular Surgery, Hunan Provincial People’s Hospital (The first-affiliated hospital of Hunan normal university), Changsha, China; ^2^ Nursing Management Department, Hunan Provincial People’s Hospital (The first-affiliated hospital of Hunan normal university), Changsha, China

**Keywords:** obstructive jaundice, Percutaneous transhepatic biliary drainage (PTBD), interventional specialist care clinic, data analysis, postoperative targeted nursing

## Abstract

Jaundice is a detection index in many disease conditions commonly characterized by yellowish staining of the skin and mucous membranes. This work studies the postoperative care outcome in 1,246 patients (669 males and 577 females) with obstructive jaundice who underwent percutaneous transhepatic biliary drainage (PTBD). These patients were admitted to the interventional vascular surgery department of our hospital from February 2017 to February 2022. From the results, frequent wound re-dressing and maintenance of the drainage tube had significant positive influence on wound healing and patient recovery. The data also showed strict adherence by patients to the doctor’s recommendation advising them to visit the interventional specialist care clinic in time for wound dressing change and drainage tube maintenance. As a result, there was no significant difference in wound allergy, exudation, redness and loosening among patients. A cross-sectional analysis of the effect of age on recovery revealed variations in the healing pattern (wound loosening and the redness) between patients of different ages although the relationship is not very clear due to the limited sample size. Efficient drainage tube maintenance promoted recovery and prevented the occurrence of related complications such as PTBD tube blockage and biliary tract infection. The establishment of the interventional specialist care clinic used in this study additionally ensures patients’ safety, and the incidence of complications have been reduced drastically. These achievements are attributable to the implementation of regular dressing change, drainage tube maintenance and health education for patients with PTBD tube. These practices have also improved on the level of specialty in nursing practice, increased the professional value of nurses and better recognition by the society.

## Introduction

The occurrence of jaundice usually diagnosed by the yellowing of skin and mucous membrane serves as a marker in many diseases with its symptoms, treatment and prognosis varying accordingly (1–4). The Statistical Package for the Social Sciences (SPSS) software has been widely applied in the analysis of jaundice related data (5), whereas pathological analysis is gradually used more frequently in jaundice works (6–8). Data analysis can be used to establish the relationship between some neonatal diseases and the rate of jaundice diagnosis (9–11). Gallbladder carcinoma (GBC) patients presenting with jaundice can benefit from resection (12–14). Another data-based research has analyzed mathematical models relevant in predicting the survival rates of patients with biliary atresia (15–17). Similarly, retrospective analysis of patients suffering from autoimmune pancreatitis (18, 19) and acute onset autoimmune hepatitis (20) has uncovered interesting conclusions about the clinical diagnosis of jaundice. These studies have contributed remarkably to the diagnosis and treatment of patients with jaundice-related symptoms and are also beneficial in motivating researchers to carry out retrospective statistical analysis preferably of large-scale data collected over a longtime span. Our review of the existing studies conducted on jaundice and its related pathologies reveal the need to conduct more research focused on analyzing the type and quality of clinical (nursing) care offered to patients with jaundice and how they influence recovery and patients’ safety.

Obstructive jaundice is a clinical disease often caused by malignant tumors (1). It is treatable by percutaneous transhepatic biliary drainage (PTBD); a procedure involving percutaneous insertion of a special puncture needle into the intrahepatic bile duct under the guidance of X-ray or B-ultrasound, followed by catheterization and drainage to relieve the symptoms of obstruction (21, 22). Rapidly visualization of the intrahepatic and extrahepatic bile duct is guided by the injection of contrast agents. PTBD can reduce serum bilirubin in patients with malignant obstructive jaundice (MOJ), which aids to restore the patients’ liver and kidney functions and improves on quality of life. PTBD also increases survival time during which different treatment options can be explored. For patients with benign obstructive jaundice, PTBD can benefit the rapid relief of symptoms of jaundice and infection, and provide access for subsequent treatment.This method was first proposed by Remolar in 1956 and has been widely employed in clinical practice ever since (23). Along with biliary stent implantation, PTBD can effectively alleviate obstructive jaundice. It has been extensively used as the preferred measure for clinical treatment of obstructive jaundice due to advantages such as ease of operation, higher success rate and relatively few postoperative complications (24, 25).

Many patients require long-term PTBD tube after surgery Therefore, standardized PTBD drainage tube care is pertinent to reduce related complications, ensure efficacy of the interventional surgery and improve patient’s quality of life (26, 27). On the basis of providing continuous care, our hospital opened the first interventional specialist care clinic in China in February, 2017.Where we perform targeted nursing techniques and implement measures such as regular wound dressing change, replacement of catheter fixation devices, maintenance of drainage tubes, and specialized health education for patients after PTBD surgery. Our team also published the work titled “Expert consensus on drainage tube nursing of percutaneous transhepatic biliary drainage” (28).

The present study analyzes the effect of postoperative specialized outpatient nursing care on 1,246 patients (669 males and 577 females) treated with PTBD. The one-way analysis of variance (ANOVA) was used to study the impact of gender on wound loosening, wound health, redness, exudation, and allergy. Also, Chi-square test (cross-analysis) was used to analyze the relationship between age and treatment outcomes. Finally, Poisson regression analysis was used to investigate the significance of dressing change and drainage tube maintenance to superior wound healing. The results obtained from this study will enrich the research status of obstructive jaundice, emphasize on the importance of quality patient centered clinical care and supplement studies aimed at data analysis of conditions related to postoperative PTBD tube care in patients with obstructive jaundice.

## Objective and Methods

### General Description

A total of 1,246 patients (669 males and 577 females) with obstructive jaundice were selected as the research subjects. These patients were admitted to the interventional vascular surgery department of our hospital from February, 2017 to February, 2022.

#### Inclusion and Exclusion Criteria

Inclusion criteria:

(1) Age: 20–83 years old;
(2) Conscious; can communicate and agreed with the data collection

Exclusion criteria:

(1) Unconscious; can’t communicate effectively
(2) For various reasons, the patients are unable go to the intervention specialist nursing department for drainage tube care
(3) Abnormal cardiopulmonary function; patient’s condition is not stable

### Treatment

All the patients were treated successfully with PTBD in our hospital and advised to visit the interventional specialist care clinic after discharge where we performed puncture wound dressing change and drainage tube maintenance. The standardized procedure included patient evaluation, drainage tube rinse, replacement of drainage device and dressing change (including replacement of catheter fixation device), handling of special cases (emergencies), medical evaluation, specialized health education and other targeted approaches. Overall, the possibility was reduced for unscheduled emergency hospitalization due to various reasons like infection of the puncture wound, biliary tract infection, accidental tube blockage and extubation caused by loosening of a catheter fixing device. Also, patients experienced less discomfort due to effective pain management and medical expenses were reduced, benefiting the sustenance of national medical resources.

### Observation Indicators

The relationship between wound healing and patient factors including gender, age and general conditions (including Stooling and urination, jaundice, fatigue and abdominal pain) was analyzed. Also, the effects of the regular interventions (dressing change and drainage tube maintenance) on the wound and drainage conditions were evaluated in outpatients with PTBD surgery.

### Time Observation

The recorded data included the clinical symptoms of patients after surgery and the recovery time.

### Statistical Analysis

Statistical analysis was performed on all experimental data using SPSS 23.0 software. Run-length analysis, ANOVA, chi-square test, correlation analysis, and Poisson regression analysis were used.

## Results and Discussion

Data screening: Originally, 1,297 patients were screened from the original data, where 1,246 patients were deemed valid with information suitable for analysis. Table 1 shows the Gender and discharge diagnosis analysis. The invalid data including patients absent from the hospital after online registration, or had missing information like age and gender were deleted. In total, 1,246 patients (577 females and 669 males) were studied in this work.

**Table 1:** Gender and discharge diagnosis analysis.

Subject	Name	Gender		Amount to
		Woman	Man	
Discharge diagnosis	Malignant obstructive jaundice	557	614	1,171
	Obstructive jaundice	7	0	7
	Hepatolith	0	8	8
	Hepatapostema	0	30	30
	Hilar cholangiocarcinoma	5	3	8
	Liver metastasis of intestinal cancer	0	3	3
	Malignant tumor of pancreas ampulla	2	0	2
	Cholangiocarcinoma	1	3	4
	Gall stone	1	6	7
	Biliary obstruction	0	2	2
	Carcinoma of head of pancreas, malignant obstructive jaundice	3	0	3
Amount to		577	669	1,246

As shown in Table 2, the run-length test was used to analyze whether the ages of the patients used in the study followed a randomly distributed data sequence. The resulting *p* value (*p* > 0.05) confirms the original assumption that the data were subject to random distribution and true.

**Table 2:** Age statistic analysis.

Name	Sample size	Statistical value z	*p*
Age	1,246	−1.384	0.166

As shown in Table 3, one-way ANOVA was used to study the effect of gender on the differences observed in wound loosening, recovered wound, wound redness, wound exudation and wound allergy among patients. The analysis reveals that the patient’s gender had no significant effect (*p* > 0.05) on the assessed parameters, suggesting that the healthy appearance and healing of the PTBD puncture site is not gender specific.

**Table 3:** Gender and wound analysis.

	Gender (mean ± standard deviation)		*F*	*p*
	woman (*n *= 576)	man (*n *= 670)		
wound loosening	1.95 ± 0.41	1.92 ± 0.42	0.572	0.450
recovered wound	10.57 ± 3.71	10.70 ± 3.54	0.195	0.659
wound redness	2.87 ± 1.43	3.17 ± 1.66	2.813	0.095
wound exudation	8.51 ± 4.06	9.02 ± 3.72	1.192	0.276
wound allergy	1.87 ± 0.41	1.93 ± 0.25	0.523	0.472

The one-way ANOVA (Table 4) was used to study the possible gender differences between observed stooling and urination, jaundice, fatigue and abdominal pain. Similar to the result obtained for the effect of gender on wound conditions, there was no significant differences in the appearance of these symptoms among both sexes (*p* > 0.05), meaning that there was consistency in the diagnosis of stooling and urination, jaundice, asthenia and abdominal pain.

**Table 4:** Analysis of variance between gender and general situation.

	Gender (mean ± SD)		*F*	*p*
	woman (*n *= 576)	man (*n *= 670)		
Stooling and urination	9.74 ± 2.24	9.70 ± 2.30	0.069	0.792
Jaundice	1.85 ± 0.35	1.91 ± 0.29	3.036	0.082
Fatigue	1.85 ± 0.36	1.92 ± 0.28	2.429	0.121
Abdominalgia	2.06 ± 0.67	2.15 ± 0.59	0.463	0.498

**Figure 1:**
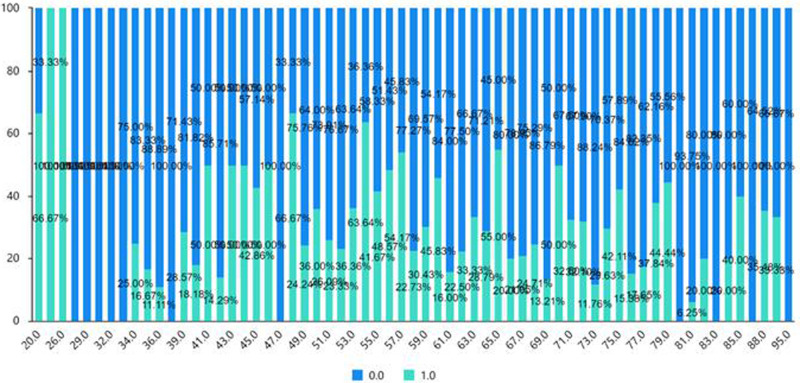
Cross-sectional view of age and wound loosening (0 = none, 1 = yes).

**Figure 2:**
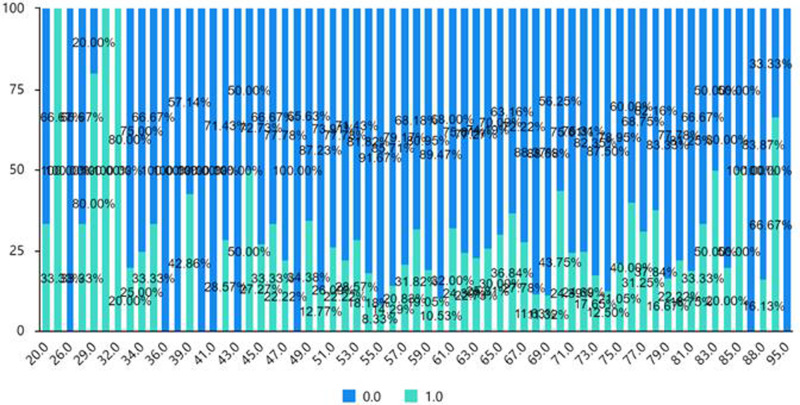
Cross-sectional view of age and redness of the wound (0 = none, 1 = yes).

The chi-square test (cross-analysis, Table 5) was used to study the differences in wound loosening, wound redness, wound exudation, and wound allergy among ages of patients. The analysis shows no significant age dependent differences between wound exudation and wound allergy (*p* > 0.05). Conversely, wound loosening and redness were significantly influenced by age (*p* < 0.05). As shown in Figures 1 and 2 (the cross-sectional view of age and patients’ conditions), age affects the wound loosening and the redness of the wound, although the relationship is not very clear due to the limited sample size.

**Table 5:** Age and wound Chi-square test statistics process values.

Item	name	value
Age * wound loose	Pearson Chi-square	105.930 (*p *= 0.000**)
	Continuously correct Yates chi-square	105.930 (*p *= 0.000**)
	Fisher chi-square	-
	E ≥ 5	69 (54.76%)
	1 ≤ E < 5	40 (31.75%)
	E < 1	17 (13.49%)
	Cnt	126
	*N*	1,231
	Df value of degree of freedom	62
Age * redness of wound	Pearson chi-square	87.095 (*p *= 0.020*)
	Continuously correct Yates chi-square	87.095 (*p *= 0.020*)
	Fisher chi-square	-
	E ≥ 5	61 (48.41%)
	1 ≤ E < 5	45 (35.71%)
	E < 1	20 (15.87%)
	Cnt	126
	*n*	1,201
	Df value of degree of freedom	62
Age * wound exudation	Pearson chi-square	64.641 (*p *= 0.385)
	Continuously correct Yates chi-square	64.641 (*p *= 0.385)
	Fisher chi-square	-
	E ≥ 5	55 (43.65%)
	1 ≤ E < 5	48 (38.10%)
	E < 1	23 (18.25%)
	Cnt	126
	*n*	1,198
	Df value of degree of freedom	62
Age * wound allergy	Pearson chi-square	53.801 (*p *= 0.761)
	Continuously correct Yates chi-square	53.801 (*p *= 0.761)
	Fisher chi-square	-
	E ≥ 5	45 (35.71%)
	1 ≤ E < 5	38 (30.16%)
	E < 1	43 (34.13%)
	Cnt	126
	*n*	1,234
	Df value of degree of freedom	62

* p < 0.05; **p < 0.01.

Correlation analysis (Table 6) was used to study the relationship between age and general conditions including defecation, jaundice, fatigue, and abdominal pain. The Pearson correlation coefficient was used to indicate the strength of the correlation. The analysis showed no relationship between age and the symptoms (*p *> 0.05).

**Table 6:** Pearson correlation analysis between age and general situation.

		Urine and stool	jaundice	fatigue	abdominalgia
Age	correlation coefficient	0.060	0.000	0.000	0.007
	*p* value	0.050	1.000	1.000	0.941

*p < 0.05; **p < 0.01.

As shown in Table 7, correlation analysis was used to study the relationship between the time interval between two hospital visits and respective wound conditions (wound allergy, exudation, redness, loosening, recovered wound). Pearson correlation coefficient was used to indicate the strength of the correlation. The findings denote a lack of correlation between the time interval between two hospital visits and the wound parameters (*p *> 0.05). This result confirms the relevance of strict compliance. and timely visits to the intervention specialist care clinic for wound re-dressing and drainage tube maintenance.

**Table 7:** Pearson correlation-detailed format.

		Wound allergy	Wound exudation	The wound was red	The wound is loose	The wound is good
Interval between two visits	correlation coefficient	−0.021	−0.032	−0.020	0.013	0
	*p* value	0.451	0.256	0.472	0.638	0.993

*p < 0.05; **p < 0.01.

As shown in Table 8, Poisson regression analysis was performed to ascertain the impact of the treatments (dressing change and drainage tube maintenance) on wound healing. The Poisson regression models were obtained as log(u) = 2.067 + 0.122 and log(u) = 2.067 + 0.001 for dressing change and drainage tube maintenance respectively, where u represents the expected mean.

**Table 8:** Summary of Poisson regression analysis results (*n* = 659).

item	coefficient of regression	Standard error	*z value*	*p* value	OR value	OR value 95% CI
Dressing change	0.122	0.012	9.936	*p* < 0.01	1.129	1.103∼1.157
Drainage tube maintenance	0.001	0.000	2.839	0.005	1.001	1.000∼1.001
intercept	2.067	0.037	56.496	*p* < 0.01	7.900	7.354∼8.488

*Dependent variable: recovered wound.*

*Independent variables: dressing change and drainage tube maintenance.*

*McFadden R formula: 0.023.*

Dressing change had a significant positive impact on wound healing as it showed significant statistics (*z* = 9.936, *p* < 0.01). It has an odds ratio (OR) value of 1.129, implying a 1.129-fold increase in wound healing with a unit increase in wound re-dressing.

Similarly, good drainage tube maintenance supported wound healing (*z* = 2.839, *p* = 0.005 < 0.01). An OR value of 1.001 indicates a 1.001-fold increase in wound healing following a unit increment in proper drainage tube maintenance.

Therefore, it is logical to recommend adequate dressing change and drainage tube maintenance to achieve wound healing and quicker patient’s recovery.

Lastly, Pearson analysis was used to study the correlation between drainage tube maintenance and normal drainage condition as well as slag entrainment during drainage (Table 9). Pearson correlation coefficient was used as a measure of the strength of the correlation.

**Table 9:** Pearson correlation-detailed format.

Treatment Opinion: Dressing change		Treatment Opinion: pipe maintenance
Normal drainage	correlation coefficient	0.089**
*P* value	*P* value	0.006
Drainage slag	correlation coefficient	−0.109*
*P* value	*P* value	0.040

*p < 0.05; **p < 0.01.

A significant correlation between drainage tube maintenance and normal drainage condition (*p* = 0.006) informs us that ensuring correct maintenance of the PTBD tube is necessary for complication-free biliary drainage.

## Conclusion

This work analyzed data obtained from 1,246 patients (669 males and 577 females) with obstructive jaundice who underwent PTBD surgery and subsequent follow up at the interventional specialist care clinic of our hospital from February 2017 to February 2022.

The results show:

(1) Dressing change and drainage tube maintenance both had significant positive influence on wound recovery.
(2) Timely (on-schedule) visits to the interventional specialist care clinic for wound dressing change and drainage tube maintenance encouraged desirable wound healing pattern and indifference in observed wound allergy, exudation, redness and loosening among patients.
(3) As discovered in the cross-sectional view of gender and age versus patients’ conditions, gender was found to have no influence on any of the wound properties while age to a significant extent controlled wound loosening and redness. Nevertheless, this effect of age on wound healing remains unclear (due to the limited sample size in our study) signifying the need for further studies to unravel the underlying mechanism(s).
(4) Finally, appropriate drainage tube maintenance was imperative to guarantee normal biliary drainage, thus avoiding related complications such as PTBD tube blockage and biliary tract infection.

We can therefore conclude that the institutionalization of the interventional specialist care clinic and the incorporation of targeted post-operative nursing care (regular dressing change, good drainage tube maintenance and health education of patients after PTBD surgery) has contributed enormously to ensuring patients’ recovery and safety, rightfully so because the incidences of complications have been remarkably minimized. The quality of overall nursing care has also improved and attracted well-deserved appreciation by the society.

## Data Availability

The original contributions presented in the study are included in the article/supplementary material, further inquiries can be directed to the corresponding author/s.
